# The repressed life of adult female patients with mild ADHD

**DOI:** 10.3389/fpsyt.2024.1418698

**Published:** 2024-10-09

**Authors:** Shigenobu Toda, Sakiko Tsushima, Osamu Takashio, Mitsuru Kikuchi, Haruhisa Ohta, Tatsuya Nagasawa, Akira Iwanami, Yutaka Ohashi

**Affiliations:** ^1^ Department of Psychiatry, Showa University School of Medicine, Tokyo, Japan; ^2^ Department of Psychiatry, Shizuoka Psychiatric Center, Shizuoka, Japan; ^3^ Department of Neuropsychiatry and Behavioral Sciences, Kanazawa University School of Medicine, Kanazawa, Japan; ^4^ Division of Human Developmental Sciences, Graduate School of Humanities and Sciences, Ochanomizu University, Tokyo, Japan; ^5^ Medical Institute of Developmental Disabilities Research, Showa University, Tokyo, Japan; ^6^ Department of Neuropsychiatry, Kanazawa Medical University, Kahoku, Japan

**Keywords:** ADHD, female, rumination, overadaptation, camouflaging, impulsivity, ASD

## Introduction

**Figure 1:**
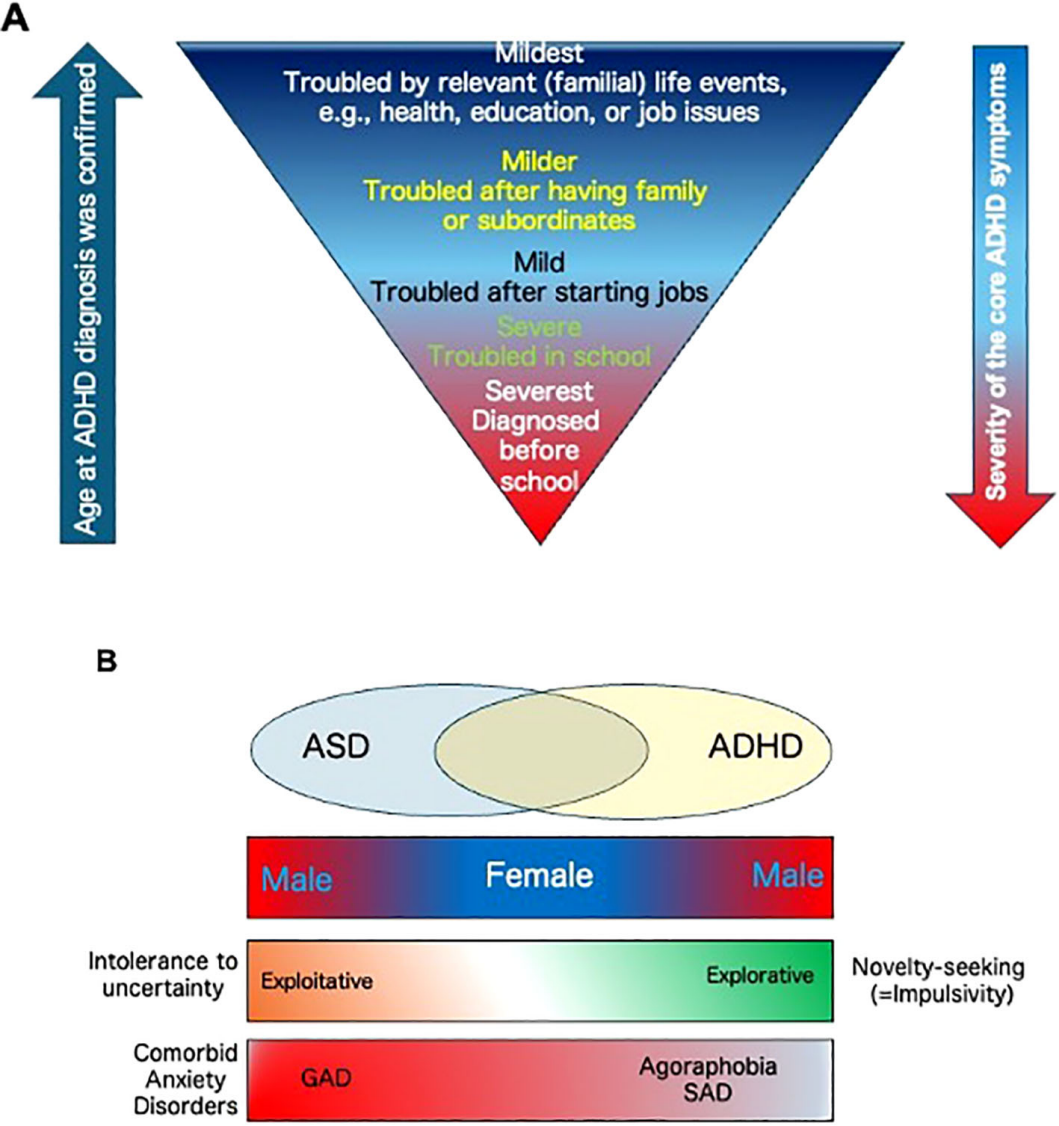
**(A)** The relationship between severity and timing of ADHD diagnosis. Patients’ problems become more context-dependent with increasing age of diagnosis, and their symptoms become less typical compared to younger severe cases. Note that there are no strict diagnosing criteria to distinguish between mild- and core ADHD patients. From the social functioning point of view, in this text, we term the patients with “core ADHD” as being severely interrupted in their daily lives both at home and outside their homes, such as schools or workplaces, by three representative and outward ADHD symptoms that include inattention, high impulsivity, and hyperactivity, which allow to make their diagnoses quickly at the center of the criteria. In contrast, we term the patients with “mild ADHD”as those whose daily activities are still somewhat compromised by the ADHD symptoms, but their effort to overcome or curb them results in success mostly or partially, at least in public. Yet, their daily lives are affected by various atypical, inward, or poorly characterized symptoms or difficulties. Because of the marginality of the criteria, their diagnoses are often difficult to make or delayed. **(B)** A working model reflecting the relationship between gender, impulsivity, attitude to uncertainty, and comorbid anxiety disorders among the core ASD/ADHD groups and their overlapping (intermediate) phenotypes.

Attention-deficit/hyperactive disorder (ADHD) is a spectrum disorder (1) whose severity of symptoms may range from subthreshold cases to the severe core group, similar to the autistic spectrum disorder (ASD) (for definition of terms, see **Figure 1** legend) (2). In general, ADHD can be easily diagnosed when patients are very young. However, patients with milder symptoms, which may comprise the majority of patients with ADHD, as shown in **Figure 1A**, may not be diagnosed—or even misdiagnosed—at an early stage, rendering their phenotypes less typical as in the case of spectrum disorders. Contrary to the widespread notorious ADHD symptoms, such as sloppiness, roughness, overconfidence, recklessness, loss of time, and inattentiveness, patients with milder symptoms are rather sensitive and very concerned about past failures and mistakes, even during childhood. Despite their relatively good reputation at their offices or schools, their self-esteem remains very low, and their extreme caution results in overadaptation.

Many physicians and researchers have begun to illuminate the significance of these symptoms from both clinical and scientific points of view (3). However, in contrast to core ADHD patients, a lack of precise feature characterization unavoidably results in underestimating or even overlooking their severity and long-term effects. Here, we, as the clinical experts treating adult ADHD at psychiatric facilities in Japan, detail their understated but distinctive nature and psychopathological phenotypes to improve this situation.

## Overadaptation at the workplace and consequential untypical symptoms in mild cases

Patients with mild ADHD may often encounter a plethora of issues when they start working. For instance, they may need to perform various tasks they have never engaged in before and must, therefore, learn how to anticipate them seamlessly, which might be their greatest challenge ever. These situations mercilessly expose their inattentiveness or carefully hidden difficulties in executive functions (1, 4, 5), such as planning, prioritization, scheduling, and multitasking. Even after their promotion, additional barriers will emerge because they will be forced to demonstrate their skills in organizing and supervising others for the first time. Concomitant consulting, listening to, advising, and instructing, attributes that are essential for managing people, require different sets of skills and abilities that are not inherent to people with ADHD or ASD. Eventually, these individuals occasionally exhibit various anxiety-, depressive- or psychosomatic signs. However, the causality between their cognitive load and maladaptation appears obvious because once their additional/unfamiliar duty is eliminated, their symptoms may rapidly ameliorate. Consequently, these symptoms can be considered as signs of adjustment disorder. This pattern is analog to what has been observed among patients with mild intellectual disability or borderline intelligence quotient. However, in the case of ADHD, symptoms are more transient, context-dependent, and primarily stem from the imbalanced abilities of these individuals.

Although it is widely accepted that both impulsivity and hyperactivity are not so pivotal in adult patients with ADHD, this does not necessarily mean that these problems have been totally overcome. When they are overloaded and/or their top-down control is disturbed, their weakness will be re-exposed, thereby increasing their monologues, losing calmness, becoming agitated, or demonstrating panic-like behaviors. Nonetheless, their innate difficulty is underestimated owing to their (ability of) over-carefulness. They are consciously or unconsciously aware that their default is so inattentive that they behave in a compensative manner, for example, by providing multiple confirmations.

After all, they are often misdiagnosed not only due to the mildness of their impairments or problems but also because of their various and more apparent comorbidities. In fact, many adult ADHD patients have undergone some psychiatric treatment at other clinics or hospitals before being diagnosed with ADHD (6–8). When the primary treatments do not work, they are then introduced to or voluntarily come to us. Typically, the chief complaints during their first visit to clinical settings are as follows:

1) Insomnia on weekdays compared to hypersomnia on weekends.

2) Stiff shoulder and tension-type headache after work.

3) Chronic fatigue.

4) Stomachache and/or nausea, especially at the workplace.

5) Loss of motivation and depressive mood.

These complaints do not appear to be ADHD or ADHD-based anguish. As such, these individuals often claim to be suffering from depression. However, these symptoms are commonly the result of being overstrained by prolonged and/or overactivated autonomic nervous system (9, 10). In addition, several characteristic and contrasting activities/behavioral patterns exist between daytime and nighttime or weekdays and weekends. For example, on weekdays, most of them have difficulties in getting up, cannot have breakfast, and even have irritable bowel syndrome (IBS) in the morning. In addition, they often suffer from severe stiff shoulder and neck pain, back pain, and tension-type headaches after leaving their offices (11).

## Camouflaging, impulsivity, and nocturnal rumination

Second, they don’t enjoy lunchtime with colleagues; if possible, they skip lunch, which will be discussed later. Consequently, they feel starving after 5 PM due to slight hypoglycemia and long-term overstrain, thereby triggering overeating and drinking habits after leaving their offices. This may also result in the heightened impulsive tendency for overbuying and overeating/drinking in ADHD (12–16). In the case of Japan, probably due to cultural differences, severe impulsive actions like drug abuse are relatively rare. Thus, these behaviors may be more accessible and acceptable as milder alternatives.

These actions resemble a “rebound” phenomenon based on their intrinsic impulsivity that is augmented by excessive suppression due to overstaining or so-called “camouflaging” at the office or school. In other words, their impulsivity is controlled, at least partially, while they are with others. However, once alone or having returned home, the camouflaging is no longer necessary, and they are immediately released from daytime stress, revealing/switching on their “real” selves.

As a result, it appears as if they live a “double life,” where the nightlife is carefully hidden from others. These excess behaviors are often repeated, like a pre-sleep quasi-ritual, when top-down control becomes weak due to relief, fatigue, and/or sleepiness as if to calm themselves down.

The camouflaging itself has been proposed as one of the representative symptoms among patients with mind ASD, especially in females (17, 18). However, this has also been frequently observed among female ADHD patients, especially when co-occurring with ASD (19). Importantly, ASD-ADHD overlapping, which has been accepted since the Diagnostic and Statistical Manual of Mental Disorders Fifth Edition (20), may result in various impulsive actions after daytime camouflaging, thereby exhibiting a stark contrast to ASD.

Besides, it is still challenging for them to fall asleep because of rumination regarding what happened during the daytime (something negative, impressive, or stunning) or a task scheduled for the next day, which would cause a psychological burden to them. Even during sleep, they occasionally dream of similar ruminative themes, translating into a state where they cannot feel relaxed and causing significant fatigue the following day (21)—the lack of good sleep on weekdays results in oversleeping on weekends. As a result, they waste their holidays, rendering them unable to change their moods for the upcoming week. The rumination itself is also not specific to ADHD/ASD (22). Still, attentional bias to something negative, difficulty in attentional switching, and the excess anxiety tendency in ADHD collaborate to elicit and maintain rumination (23–26).

## Avoiding situations that are not relaxing

Their inability to enjoy lunch or party with colleagues or crowded parties is another characteristic feature of mild ADHD. Although these individuals may enjoy spending time with one or two familiar people, their levels of coping or inconvenience drastically increase as the number of people increases or when someone unfamiliar joins, such as a supervisor. This is not only explained by their comorbidities, including social anxiety disorder (SAD) and/or auditory hyperesthesia. It must be highlighted that it is significantly challenging for these individuals to shift the focus of their attention from one to another while chatting and eating with many people because it is difficult for them to predict who will speak next. In addition, these individuals must keep what others have talked about in their minds, at least for a while. Furthermore, they also must join the conversation. Thus, besides their social phobia, having lunch or attending parties can be a significantly challenging multitasking cognitive task, which requires working memory, prediction, reading of unpredictable conversation flows, and attentional switching/allocation/selection, rendering this a far more complex situation than those experienced in their regular duties (27–29).

## Comorbidities and outlets for impulsivity

Comorbidities are another severe issue for adult ADHD patients. This may be more prominent among female than male ADHD patients (30, 31). Anxiety disorders are one of the most common comorbidities in ADHD. Generalized anxiety disorder (GAD) is the most notable type of anxiety disorder, followed by SAD or agoraphobia (32–35). In addition, those with co-occurring ASD and ADHD display more levels of anxiety than those with either ASD or ADHD (36).

Even when the diagnosis of anxiety disorders is subthreshold, there are many common minor symptoms between ADHD and anxiety disorders, such as rumination, hyperarousal, overstrain, hypersensitivity, and insomnia. Interestingly, they are also characteristic of GAD (33). The manifestation of these somatic symptoms is milder on weekends, displaying another contrast from weekdays.

In general, both core groups of ASD and ADHD mainly consist of male. Meanwhile, female patients comprise the milder intermediate group in terms of ADHD and ASD severity. Instead, the female intermediate group possesses more ASD-based anxiogenic properties (37, 38), and various factors may be attributed (39). Among them, intolerance to uncertainty in ASD would be particularly of interest when novelty-seeking of ADHD is located on the opposite side of the same “uncertainty” axis (40) (**Figure 1B**).

Both bipolar disorder and major depressive disorder are prevalent comorbidities of ADHD (41, 42). Of note, ADHD is often accompanied by repeated and/or atypical depression featured by oversleeping (43). Atypical depression may frequently be seasonal or deteriorate during the rainy season. Some reports revealed the seasonal alteration of the severity of ADHD symptoms (44, 45). People with ADHD often claim to be dazzling under the sun, which reflects their hypersensitivity to the sunshine (=photophobia; 46). This may, in turn, be related to mood instabilities under limited sunshine or day-night reversal conditions (47). The overlap between ADHD symptoms, seasonal depression, and delayed sleep phase syndrome has also been reported (48).

In addition, there are various somatic comorbidities among ADHD patients, including asthma, rhinitis, hypertension, atopic dermatitis, IBS/crone’s disease/ulcerative colitis, obesity, diabetes, and sleep apnea syndrome (49–51). Many may be commonly attributed to hypersensitivity to changes in internal/external environments and could be aggravated by automatic nervous system overactivation (9).

Lastly, females with mild ADHD tend to seek specific social activities more exploratively compared to the core ASD group. For example, in Japan, many female ADHD students join extracurricular activities such as brass bands, cheerleading, or acting classes. These activities are characterized as group-based, physical, or musical, suggesting the intrinsic preference that may healthily satisfy their impulsivity and hyperactivity, releasing them from their regular constraints at school or switching their attention from ruminative worries. Unfortunately, however, these opportunities are often lost once school is finished, subsequently becoming full just for going through their daily duties without affording relaxation or socializing. It, therefore, comes as no surprise that during the COVID-19 era, individuals with mild ADHD who were deprived of these opportunities reached their limits, became desperate, and even committed self-injuries or suicide because of unreleased impulsivity and augmented rumination and anxiety (52–54).

## Concluding remarks

In this opinion, we focused on the overlooked or underestimated characteristic features that are very specific to adult females with mild ADHD. They may be culturally affected and vary under different environments. However, we believe there are fundamental similarities underlying them, and they may help avoid underdiagnosis or even misdiagnosis due to comorbidities of ADHD in adults to ease their daily difficulties.

The daily lives of females with mild ADHD demonstrate a stark contrast between daytime and nighttime or weekdays and weekends, which is far from ordinary. complicated, intractable, and vulnerable nature appears pathetic and requires significant attention and greater understanding.
